# Fair relationships and policies to support family day care educators’ mental health: a qualitative study

**DOI:** 10.1186/1471-2458-14-1214

**Published:** 2014-11-25

**Authors:** Lara Corr, Elise Davis, Kay Cook, Elizabeth Waters, Anthony D LaMontagne

**Affiliations:** Jack Brockhoff Child Health and Wellbeing Program, Melbourne School of Population and Global Health, University of Melbourne, Level 5, 207 Bouverie Street, Carlton, Victoria 3010 Australia; Centre for Applied Social Research, RMIT University, Melbourne, Victoria 3001 Australia; Population Health Strategic Research Centre, School of Health & Social Development, Deakin University, Burwood, Melbourne, Victoria 3125 Australia; Centre for Health Equity, Melbourne School of Population and Global Health, University of Melbourne, Level 5, 207 Bouverie Street, Carlton, Victoria 3010 Australia

**Keywords:** Child care, Mental health, Occupational health, Policy, Child care provider

## Abstract

**Background:**

High quality child care is a population health investment that relies on the capacity of providers. The mental health and wellbeing of child care educators is fundamental to care quality and turnover, yet sector views on the relationship between working conditions and mental health and wellbeing are scarce. This paper examines child care educators’ and sector key informants’ perspectives on how working in family day care influences educator’s mental health and wellbeing.

**Methods:**

Semi-structured telephone interviews were conducted with Australian family day care educators (n = 16) and key informants (n = 18) comprised of representatives from family day care schemes, government and other relevant organisations regarding the relationship between working conditions and educator mental health. Thematic analysis referenced the assumptions and concepts of critical inquiry and used social exchange theory.

**Results:**

Educators and key informants reported that educators’ mental health was affected by the quality of their relationships with government, family day care schemes, and the parents and children using their services. These social relationships created and contributed to working conditions that were believed to promote or diminish educators’ mental health. High quality relationships featured fair exchanges of educator work for key resources of social support and respect; adequate income; professional services; and information. Crucially, how exchanges influenced educator wellbeing was largely contingent on government policies that reflect the values and inequities present in society.

**Conclusions:**

Making policies and relationships between educators, government and family day care schemes fairer would contribute strongly to the protection and promotion of educator mental health and wellbeing, and in turn contribute to workforce stability and care quality.

## Background

Early childhood settings are critical sites in which to promote the mental health of child care educators as workforce sustainability and quality care are inextricably linked to educator mental health and wellbeing[1–3]. There is a compelling case for addressing worker mental health; the prevalence of mental illness in employees is increasing, as is its cost to workplaces, economies, workers and societies[4]. A positive relationship between work quality and mental health is now well established, along with the importance of securing workers’ legal and ethical rights to psychologically safe work[5, 6]. The prevention of poor mental health and the promotion of mental wellbeing represent critical opportunities to improve population health[7]. Hence, the emerging field of mental health promotion, broadly defined as "any action to enhance the mental well-being of individuals, families, organisations or communities"[8], is now understood to be important for individual, community and societal functioning[9]. Understanding how mental health is protected, promoted or diminished is central to developing mental health promotion initatives.

This article focuses on understanding how educator’s mental health is influenced by working conditions in family day care, from the perspective of educators and sector key informants. Family day care (FDC) is a common form of child care internationally[10] that is used by approximately twelve per cent of families in Australia[11]. It is home-based, formal child care located in the educator’s home and attended by mixed-aged groups of pre-school children as well as older children during non-school times (e.g. before and after school) and periods (e.g. holidays). In Australia approximately 18,000 FDC educators care for around 125,000 children annually[11, 12]. The majority of FDC educators are registered as self-employed, ‘sole traders’ with the Australian Tax Office and have their own Australian Business Number (ABN) (pers comms, Family Day Care Australia 2014). Each educator that operates FDC as a small business is required to be independently contracted to a government accredited FDC coordination ‘scheme’. Government regulation and the payment of levies to schemes by educators dictate that schemes provide a range of services to educators. Services include monitoring in regard to regulations and standards, as well as professional support during monthly home visits or via telephone/email, processing of government child care rebates, filling child care vacancies, professional development, support in emergencies and in some instances access to toy libraries, playgroups and educator counselling. Educators are bound both by government regulations and the policies and processes of their affiliated schemes. They occupy a grey space between contractor and employee, which has implications for their autonomy, entitlements and working conditions. The relationship between working conditions and educator mental health has been identified as an important and under-researched issue for FDC, with implications for turnover and care quality[13].

Quantitative research in the field of occupational health has demonstrated that certain psychosocial and structural working conditions lead to job stress and mental illness[14, 15]. However, it has focused less on gathering context-specific evidence to develop tailored job stress and mental health promotion interventions[16]. Such intervention development work has been described as vital to create effective, comprehensive and tailored mental health promotion[17]. Community intervention researchers have found that an understanding of ‘context’ – such as within specific workplaces or sectors – is critical to the success of an intervention[18]. Before such interventions can be developed, qualitative work is required to gain an understanding of workplace contexts and the specific factors that promote or diminish mental wellbeing.

To address this critical gap, research must include the experiences and perspectives of workers themselves[19–21]. It must also reference key informants’ views as they have varying degrees of control over the context and can provide insights into appropriate levers for change[22, 23]. Worker and key informant perspectives are best placed to inform workplace health promotion when they ‘generate or work with existing theory’ to identify concepts and issues requiring action[24]. Thus a theoretically referenced, qualitative approach reconciles existing quantitative research and theory with lived experiences. In doing so, it adds rich contextual insights and also reveals underlying social patterning and forces, contributing vital information to the development of tailored interventions[25, 26].

Social exchange is an influential concept in economics, sociology and psychology that concerns how resources are exchanged and distributed through social relationships (see[27–29]). This concept of exchange in human relationships provides the basis to a family of theories that describe how, why and to what end, symbolic, emotional and material resources are exchanged between one person or collective and another[30]. A sociological perspective of social exchange theory highlights the importance of *fairness* in the exchange of resources that occurs between social groups[31]. This perspective highlights how differential power and group characteristics influence which groups have their needs met and which groups do not. In the work context, social exchange theories provide an explanation as to how the perceived fairness of working conditions can support or diminish worker’s physical and mental health[32]. The relationship between work and the mental health of workers has been tested using a quantitative measure of psychosocial working conditions based on social exchange theory, the Effort Reward Imbalance (ERI) model. ERI research predicts that when workers feel their efforts are not fairly rewarded by others through appropriate social exchange, adverse mental and physical health outcomes follow[32, 33]. Resource exchanges at the ‘micro’ level between workers and other actors can be further analysed with respect to their interactions with ‘meso’ (organisational) and ‘macro’ (societal) factors that create and perpetuate exchange conditions[34]. The influence of unfair exchange on mental health is likely to be most pronounced for workers in undervalued or low status occupations with limited power to change their working conditions; and one such occupation is child care[35].

Despite the importance of child care educator mental health and wellbeing, this subject has received little attention in the academic literature. Research into educator mental health and wellbeing has been investigated in around 16 studies (n = 19 articles). A systematic review of these studies[36] concluded that stress was a prominent issue for the childcare workforce in Australia, Netherlands and USA, where the research was located[37–44]. The review also found that the reported prevalence of depression in educators ranged widely from 6 to 38 per cent[1, 13, 45, 46]. However, as the prevalence data arose from research into American educator populations in long day care (centre-based) and FDC, it is difficult to transfer these findings to educators in other settings due to the differing social and policy contexts. Depressive symptoms were found to be related to low job resources (emotional rewards) and high work and family life ‘interference’[13, 45]. Educator mental wellbeing was investigated in two studies (Netherlands, USA), where it was found to be high and consistently related to working conditions, particularly social support[13, 47]. While these findings begin to describe mental health and its determinants in ECEC, it is worth noting that contextual differences, design limitations and reporting biases in many of the studies limit the strength of these conclusions.

More broadly, the peer-reviewed literature has reported on some of the challenging working conditions that child care educators are exposed to, such as; low pay, high responsibility and being undervalued by society[35, 48]. Studies have also highlighted issues of stress, burnout and turnover[49], yet working conditions and educator mental health and/or mental wellbeing are rarely explored together and never extensively[1, 45, 50].

### Objectives

This study aims to examine child care educators’ and sector key informants’ perspectives on how working in family day care influences educators’ mental health and wellbeing.

## Methods

This research employed a critical, self-reflective approach to investigate educator mental health in the FDC context. It involved academics, community members (i.e. FDC educators) and a range of relevant informants from the early childhood education and care (ECEC) sector. Value was placed on the knowledge and abilities that each group brought to examining the social problem highlighted by community[22]. The topic for this study emerged through conversations with FDC educators, scheme management and Family Day Care Australia (FDCA) during fieldwork for a randomised controlled trial aiming to support the mental health and wellbeing of children in FDC[51]. Through discussions over months of fieldwork, it became clear that there was a strong, shared concern about job stress, turnover and educator wellbeing by educators, scheme management and sector organisations. There was interest in how to ensure sustainability of FDC through better supporting the mental health and wellbeing of educators. In response to concern and interest, this study to collect and analyse the perspectives of FDC educators and key informants was designed and carried out in close collaboration with the sector.

Educators and FDCA contributed to discussions about appropriate phrasing of interview questions. It was agreed that questions should concern the ‘best’ and ‘hardest’ parts of working in FDC and how key actors in the lives of educators could help educators ‘feel good’ at work. Participants were aware that the study was focused on educator ‘emotional wellbeing’: the term ‘mental health’ was avoided due to its stigmatised connotations with mental illness. Given this, questioning on the ‘best’ and ‘hardest’ parts of FDC work and what could be done by different social actors/institutions to help educators ‘feel good at work’ was chosen to start discussion of how work promoted, protected or risked mental wellbeing.

Individual semi-structured interviews were conducted with 16 FDC educators and 18 key informants, in order to explore their experiences and perspectives of FDC work. Telephone interviews were the most appropriate data collection method as they could efficiently capture experiences and perceptions, and also circumvent the practical constraints of interviewing time poor and geographically scattered educators and key informants throughout two states in Australia. Although non-verbal cues are absent during telephone interviews, this method enabled greater participation and for interviews to occur in a private, familiar environment. Prior to the interviews, the primary researcher emailed or mailed a hard copy of the participant information and consent forms to educators and key informants. When the interview time was arranged, initial consent to participate was provided. Then, prior to the interview, time was taken to ensure that participants were comfortable with the researcher and the study: the researcher described the study and its motivation, the intentions for data use, participant confidentiality, data storage and offered to answer any questions. Following these discussions, informed consent was gained and demonstrated through participants signing and returning the completed participation form to the researcher. Permission was requested to record the interviews to aid in the accuracy of transcription and all participants approved this request. Ethics approval was granted by the University of Melbourne Human Ethics Committee (HREC 1034554.1).

### Sampling and recruitment of participants

The majority of FCD educators in Australia are registered with FDCA- the peak body and major insurance provider for FDC. A random sample of eight schemes in two Australian states, Victoria (n = 4) and Queensland (n = 4) (private and not-for-profit) was drawn by FDCA. These states were chosen to represent FDC at the beginning (Victoria) and more established (Queensland) stages of National ECEC reform in terms of changes such as fee deregulation and obtaining new mandatory formal qualifications. FDCA distributed an email invitation on behalf of researchers and telephoned the respective schemes to ascertain their interest in participating. Author 1 then telephoned interested schemes, who contacted educators. Educators were recruited through three avenues: 1) direct response to researcher following email invitation, 2) recommendation of a colleague (snowball sampling), or 3) scheme management invitation. As researchers did not have educator contact information, reasons for not taking part could not be collected. Additional educators were not sought for interviewing after data saturation was reached[52], which was defined as no new data arising to describe connections between working conditions and mental health and wellbeing.

Given their unique vantage points of the interface between FDC educators, schemes and policy, sector key informants were also interviewed[53]. Informants were also located in the states of Victoria and Queensland, Australia and represented a range of organisations regularly interacting with FDC educators. They were selected based on a socio-ecological framework[54] that views individuals at the centre of different ‘layers’ of influence and interaction. Educators represent the centre of the framework and can provide the greatest insights into how FDC work and working conditions influence their mental health and wellbeing as their perceptions arise from their own lived experiences. The first layer outside of individuals is referred to as the ‘micro’ level (family, peers), followed by meso (schemes, ECEC organisations) and macro levels (government, society)[55]. Representatives from the ‘meso’ level (schemes, educator and scheme professional associations, FDCA, a union and a training organisation) and the ‘macro’ (government) level of the framework were invited to participate. All informants invited to participate had a relationship with educators and could provide insight into FDC educators’ mental health and working conditions and how their organisation interacts with and views educators and FDC. These informants observe, influence or participate in creating and maintaining educator’s working conditions. Scheme management and field workers have considerable insight into FDC educators’ mental health, work and working conditions due to their role in supporting and monitoring educators. FDCA, in their role as advocates for FDC to state/territory and federal government have an indirect influence on working conditions in FDC. Representative organisations involved in training FDC educators and the FDC associations have insight into educator mental health and the difficulties that some educators experience in FDC due to their roles in providing support to educators. Though most educators are not employed by schemes, a union was interviewed as some educators contracted to local governments are part of the union and it has extensive experience in examining the fairness of working conditions for workers. Although their interactions with educators are important, parents have the least control over educators’ working conditions and little knowledge of FDC systems and pressures; therefore they were not invited for interview. Further, as parents are clients, educators engage in emotional labour to mask true emotions from them, which may make parents’ accounts unreliable[56].

Interviews were conducted from September 2011 to January 2012 and ranged from 20 minutes to 2.5 hours. During interviews with educators, the interviewer took an ‘empathetic stance’ and was positioned as a reflexive partner committed to improving the working lives of educators in FDC[24, 57]. Despite empathetic positioning as an ally, the critical approach taken in this project drew analytic attention to the social differences between participants and the interviewer and ensured that structural inequalities were not rendered invisible during the coding process[58]. All interviews were individual with the exception of two joint key informant interviews that each included two colleagues, at the request of participants. In order to allow for comparison of perspectives, the semi-structured interview guide was similar for educators and key informants. All interviews were digitally recorded and transcribed verbatim.

### Data analysis

This paper focuses on the working conditions that educators and key informants believe improve, maintain or compromise FDC educator mental health and wellbeing. The research used a critical inquiry lens, whereby ‘commonly held values and assumptions’ and ‘conventional social structures’ are interrogated throughout the research process and the research ultimately aims to contribute to social change[25]. The abductive research strategy used meant that data analysis focused on social actors’ ‘language, meanings and accounts in the context of everyday life’[59]. Data immersion occurred through listening to, transcribing and reading interviews[60]. Illustrative quotes revealing new information about how educators’ mental health is influenced by working conditions in FDC and representing ‘rich points’ were coded[61].

Rich points revealed aspects of FDC work that were perceived to be important to educators’ mental health and could not immediately be made sense of by the researcher. These points were then connected, using abductive analytical strategies[60], to the perspectives of participants, to theory and to the literature until they were understandable in the given context[61]. Abductive strategies can involve theory generation from actor’s accounts of their lives or be understood using existing theories[59]. In this instance, educator and key informant accounts have been represented using social exchange theory. The process of data analysis was non-linear and involved moving back and forth between the data, theory and literature to understand educators’ and key informants’ perspectives. The analytical tools used to explain various rich points became the key themes around which data were coded. An example of such coding concerning working conditions that protected or promoted educators’ mental health and wellbeing included minor themes of ‘social support’, ‘appreciation’, ‘respect’ and ‘positive interactions’ that were combined under the major theme of ‘positive relationships’ and connected to the rewarding nature of positive relationships in social exchange theory.

As the working conditions described took place in the context of relationships with children and families, FDC schemes and government, these three categories were used to organise results in a socio-ecological framework. The framework was important as the relationships that educators had with government, schemes and FDC clients had direct and indirect influences on educators’ mental health and wellbeing through the creation or enactment of working conditions. Government decisions concerning FDC working conditions played out through interactions with both schemes and the clients that use FDC, however schemes and clients also independently influence the development and enactment of other working conditions that may promote or risk educator mental health and wellbeing.

The final themes used in the analysis were checked and discussed with educators and scheme staff through an interactive workshop and informal discussion (i.e. member checking). Generic identifiers (e.g. Educator X, Key Informant Y) were assigned to participants, excluding demographic and organisational details to protect confidentiality within this discrete community. This study adheres to the RATS guidelines of reporting qualitative studies (http://biomedcentral.com/authors/rats).

### Sample

All but one participating educator (n = 16) was female, reflecting a highly feminised workforce. Participants were on average, 44 years old (range 20–63), again broadly representative of the general population of educators who are on average 43 years old (pers comms Family Day Care Australia, 2013). The range of time that educators had been in FDC was between 1 and 29 years (mean 8.5). Working hours ranged from 45 to 84 hours per week (mean = 63).

Educators were self-employed contractors to schemes that were either private (n = 9) or part of larger not-for-profit organisations (n = 7), i.e. faith-based social welfare organisations or local government (see Table 1). Half of the educators had been contracted to more than one scheme in their career (range 2–4 schemes). Half of the schemes provided access to toy libraries, equipment loans and play groups. Educators could set their own fees in 50% of schemes, which is optional and depends on individual schemes agreeing. Participants cared for between 4 and 30 children each week (mean = 11) with a range of characteristics: 50% (n = 8) of educators cared for children with disabilities or developmental delays; one cared for children from non-English speaking backgrounds; and three cared for children from Aboriginal or Torres Strait Islander backgrounds. Two educators were born outside Australia.

**Table 1:** **Selected sample characteristics of participating educators**

Scheme sponsorship	State	Recruitment method	Code
**Private**	Victoria	Direct contact with researcher	E1
**Private**	Victoria	Scheme	E2
**Private**	Victoria	Scheme	E3
**Private**	Victoria	Scheme	E4
**Private**	Victoria	Scheme	E5
**Private**	Victoria	Scheme	E6
**Private**	Queensland	Scheme	E7
**Private**	Queensland	Scheme	E8
**Private**	Queensland	Scheme	E9
**Not-for-profit**	Victoria	Direct contact with researcher	E10
**Not-for-profit**	Victoria	Scheme	E11
**Not-for-profit**	Victoria	Scheme	E12
**Not-for-profit**	Victoria	Scheme	E13
**Not-for-profit**	Victoria	Scheme	E14
**Not-for-profit**	Victoria	Scheme	E15
**Not-for-profit**	Queensland	Direct contact with researcher	E16

Key informants (n = 18) had varied career backgrounds; however, 13 had worked in the FDC sector and five had worked as a FDC educator. Organisations represented included private and not-for-profit FDC schemes (directors and coordination staff), state government departments, a training provider, FDC educator and scheme organisations, FDCA and a union that represents FDC and centre-based early childhood educators (see Table 2).

**Table 2:** **Selected sample characteristics of key informants**

Organisation	Position	Interview type	Code
**Family day care association, educator focused**	Leadership	Individual	KI1
**Family day care association, educator focused**	Leadership	Individual	KI2
**Family day care association, educator and scheme focused**	Manager	Individual	KI3
**Family day care association, scheme focused**	Executive	Individual	KI4
**State government**	Senior management	Individual	KI5
**State government**	Senior advisor	Individual	KI6
**Family day care peak body**	Manager	Individual	KI7
**ECEC professional development provider**	Program manager	Individual	KI8
**ECEC quality agency**	Management, frontline	Paired	KI9
**Union**	Representative	Individual	KI10
**Scheme, not-for-profit**	Manager	Individual	KI11
**Scheme, not-for-profit**	Fieldworker	Individual	KI12
**Scheme, not-for-profit**	Fieldworker	Individual	KI13
**Scheme, not-for-profit**	Managers	Paired	KI14
**Scheme, private**	Manager	Individual	KI15
**Scheme, private**	Manager	Individual	KI16

## Results

**Figure 1:**
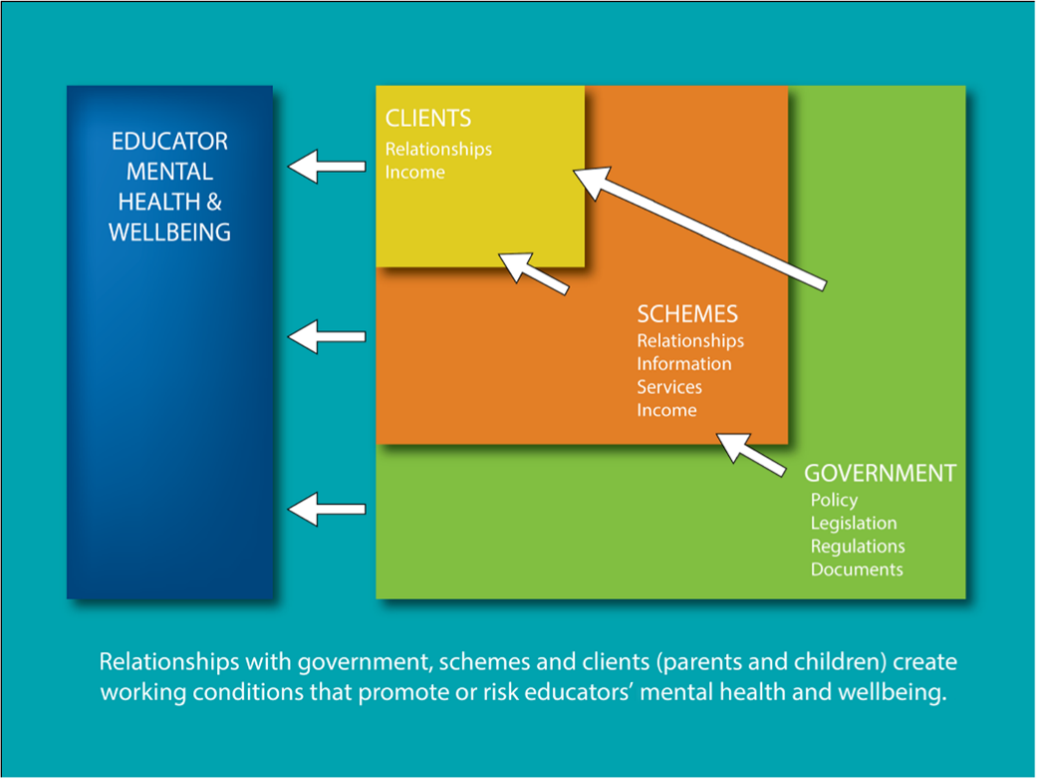
**Conceptual model of how relationships with government, schemes and FDC clients (parents and children) create working conditions that promote or risk FDC educators’ mental health and wellbeing.** This figure illustrates the direct and indirect influence of government, schemes and FDC clients on the mental health and wellbeing of FDC educators through the creation or enactment of working conditions. Government decisions concerning FDC working conditions play out through interactions with both schemes and the clients that use FDC, however schemes and clients also independently influence the development and enactment of other working conditions that may promote or risk educator mental health and wellbeing.

In presenting our results, we describe and explore accounts of what educators perceive to be the ‘best’ (support or increase mental wellbeing) and ‘hardest’ (stressful, diminishing mental wellbeing) parts of FDC work. Key informant perspectives are included throughout to demonstrate shared and unique views of how FDC work influences educator mental health and wellbeing. This binary organisation of results allowed us to draw out the nuanced relationship between meso and macro social structures of the ‘fairness’ of (Australian national) Early Years’ policies and working conditions and how they are perceived to impact on educator wellbeing. The results are separated into three key relationships in educators’ working lives that represent layers of influence on educator mental health and wellbeing (see Figure 1): the most proximal and modifiable relationship was with parents and children (FDC clients); followed by relationships with FDC coordination schemes (schemes) that were moderately close and modifiable and lastly relationships with government that affected all layers and were the most distal and challenging to alter. All relationships heavily influenced educator mental health and wellbeing, both through the quality of the relationships and the variety of working conditions they co-created, enacted or maintained. Aspects of the educators’ own working lives that they felt promoted their mental wellbeing were not a focus of this paper, however working from home, flexibility and availability for their own children and the alternative side of these positives which included long, non-standard hours, isolation and their family not seeing FDC as a ‘real job’ were mentioned during interviews.

The following results will proceed from clients, to schemes and lastly, government.

### Clients: parents and children using FDC

FDC services are important for families as they provide care and early education for children attending and provide parents with potential support and the opportunity to work, study or take respite. Educators have direct relationships with children and parents using their FDC services that can span over many years of the child’s life from infancy to school age. Overwhelmingly, educators described the joys of working with children, watching them develop and building rewarding relationships. They spoke warmly about ‘their children’ and how working with children made them feel good. "I think that’s why you love it, because you do fall in love with the children" (Educator 5). However, close relationships with children also led to significant stress when the children were considered ‘at risk’. Participants discussed ongoing stress from caring for children who were not regularly bathed; their clothes unchanged for days at a time and sent to FDC without food. In addition, educators are mandated to report suspected child abuse, which was very distressing:

[I had] two gorgeous little kids… but they were badly abused and there were all sorts of interesting explanations for the [injuries] and things like that. Just, it’s just *horrible*. So there we are, that’s the downside. Of feeling helpless when something like that happens (Educator 4).

Educators often feared for the children’s safety and after reporting suspected abuse children were often removed from FDC, compounding educator distress.

Whilst positive relationships and working with children were a clear highlight of FDC work, interactions and relationships that developed with parents over years were also important to wellbeing:

"Having such a good relationship with all of them [parents and children] is… I am very blessed to have that. It’s really good. It’s very personal how we treat each other… We are all like family" (Educator 14).

For most educators, these relationships were considered positive; however blurred personal/professional boundaries were problematic for some and on occasion had led to parents asking for inappropriate favours and problems with collecting payment.

Educators felt their mental health and wellbeing were supported by parents expressing appreciation for their work and demonstrating respect for their time and space by picking up children on time and refraining from ‘de-stressing’ by downloading their problems onto the educator at the end of the day. An educator recounted the actions of an appreciative parent:

Well there’s one parent… about [every] two or three months she buys me a bunch of flowers and I ask her why, it’s not my birthday or Christmas. She said "I don’t need an excuse to buy you flowers". And they always buy me birthday presents and Christmas presents… it makes you feel good and you feel that they appreciate what you do (Educator 12).

However, these positive relationships with parents could be undermined by unpaid or late payment of child care fees, which lead to financial insecurity and considerable job stress. As FDC incomes are generally low and precarious, these additional pressures are particularly challenging to mental wellbeing. Educators in this study were not directly questioned about their incomes, nonetheless they raised income-related issues such as low hourly rates of pay (note interviewee average of working 63 hours/week), not being able to afford to take time off work, being ineligible for small loans and being able make more money in low income jobs such as cleaning or working in a supermarket. Data is not available on FDC average wages in Australia, however median weekly incomes for full-time educators in long day care are $730, which is $423 less than the median weekly earnings for all occupations in Australia[62].

In Australia, a federal Child Care Rebate and Child Care Benefit (CCB) system means that parents are paid directly or reimbursed (each parent chooses their preferred option) a minimum of 50% of out-of-pocket costs for formal child care up to $7500 per calendar year[63]. Higher subsidies are available for parents on low incomes or undertaking job training or study. A key informant explained a reasonably common scenario:

…the family doesn’t want to pay and we could be talking over a thousand dollars but they’ve still got the [direct payment of the child care subsidy] from the government… I’ve asked [the government] and they say it’s up to us because it’s our business, to get the money back (Key Informant 18).

Educators explained that they had no indication of when a parent’s child care subsidy payment would end or if parents had ‘bad debt’ with other FDC homes. There was little support in recovering costs and no educators had been successful in retrieving larger amounts of money owed to them, despite some engaging debt collection services. One educator, who had a fairly typical arrangement whereby the scheme collected fees on her behalf, explained that she would stay with her current scheme, despite other problems, so that she would not have to collect fees directly from parents and be exposed to debt risk. As this indicates, schemes can play a crucial role in buffering job stressors and promoting the wellbeing of educators, as will be explored further in the following section.

### FDC coordination schemes

Schemes are positioned between government, parents and educators. They have a direct influence on educator mental health and wellbeing through their role in providing professional support and services.

When service provision was considered adequate by educators in the study, it was seen as being protective or supportive of their mental wellbeing. When service needs were not met, job stress arose and mental wellbeing was compromised. All educators received home visits from scheme staff; however, there was variation in educator preference of the frequency of visits and tolerance of unplanned ‘spot check’ visits. For several educators, financial insecurity arose from problems with inaccurate, or late processing of timesheets by schemes (for those whose scheme controlled pay) or from unfilled child care vacancies that educators often had limited control over as many are not allowed to privately advertise to fill spaces. Educators wishing to have unfair treatment or inadequate scheme service delivery remedied spoke of self-silencing to avoid risking essential service delivery by schemes: "A lot of my friends don’t speak a lot of English and they’re scared to speak up… they’re really scared to speak up because what happens, once you speak up the field workers don’t give you any kids" (Educator 2).

Educators and key informants described how schemes fostered mental health promoting relationships through respectful, supportive interactions. Scheme key informants emphasised educator-scheme interdependence, working in partnership with, and valuing educators: "Well without them [FDC educators, we] don’t have a job either… we need to respect what they’re doing and in order for them to respect us we’ve got to respect them so we’re just as important as each other" (Key Informant 6). Scheme management and staff felt that value and respect for FDC was exhibited through positive interactions such as celebrating the achievements of educators, including attending graduations, and acknowledging educator time and effort required to meet the increasing quality standards of FDC. Positive relationships also included an element of supervisory social support: "They [educators] can actually ring and say that they’re having a bad day and we’ll [scheme staff] talk them through it, not counselling but listen to them and debrief with them. I suppose that’s helping them mentally as well that they’re not stuck in their own little world either" (Key Informant 5). It was often mentioned that educators sought professional ‘back up’ and personal support from scheme staff and described how this made for better work quality for educators and happier educators. Positive relationships were vital buffers against difficulties faced by many educators in FDC concerning adaptation to new regulations and working with challenging children and families. There were educators who described with warmth their positive, supportive relationships with their scheme coordination staff: "Our particular coordinator now, she just fit[s] in so well with everybody and she makes everybody feel like they’re important, not just part of the system" (Educator 4). Educators drew attention to features of a high quality relationship with their scheme, such as staff showing interest in the educator, interacting in a warm and respectful way and demonstrating an understanding of the challenges and rewarding aspects of FDC. Patience and clarity in working with new regulations was also highly regarded.

Educators reported that scheme staff responses to their phone calls or emails requesting information varied. Some were considered prompt and helpful "I find them really good and easy to get along with. If I ever have a problem I can talk to them about it or I can just ring them up and ask them advice and they always help me, give me ideas of what to do" (Educator 12). Other educators found schemes frustratingly unresponsive, despite repeated attempts to seek support. It was not only receiving information that was important but also how was it delivered. Threats and alarmist communication concerning new regulations and associated fines for failing to comply were frequently recounted: "The emails saying what we need to do and that we have to do them now, this week, are very… they’re almost rude, very demanding, very ‘this is what we have to do and if you don’t do it you’ll have to pay for it’" (Educator 7).

Although educators are small business owners, a combination of scheme management style (favouring scheme control or educator autonomy) and ECEC regulations can mean an educator has limited power in how their business operates. The schemes have control over educator autonomy and how regulations play out in daily FDC operation through policies, home-visits and responding to enquiries and requests. Analysis revealed that scheme management and staff encouraged different levels of educator autonomy. Overall, private (for-profit) scheme informants appeared to view educators more as autonomous, independent business owners but provided less services and contact, whereas not-for-profit schemes treated educators more as employees and provided more support and services. Lack of autonomy caused frustration and compromised educator’s authority over their business decisions. One example was when an educator needed scheme approval for the simple business decision of providing additional FDC work to a family that needed unplanned child care. "They keep saying "It’s your business, run it the way you see fit", under the guidelines of course, but then you’ve got to ring them up and ask them if you can do overnight care or weekend care or public holiday care or whatever" (Educator 10). This experience was supported by a key informant and former FDC educator who noted "…on one hand you’re self-employed or you’re contracted to a service but on the other hand there are lots of regulations and policies and procedures that determine how you operate, and some might say might hinder your operation" (Key Informant 3). The contrast between being beholden to schemes, yet ultimately alone in bearing the risks of running a small business was difficult to reconcile. "There is a long list of policies and regulations to follow but if something goes wrong you’re on your own, self-employed" (Key Informant 9). Despite a shared foundation based on government policy and regulations, there was variation in how schemes managed these parameters, their service provision and therefore the perceived success of educator-scheme relationships.

### Government

Although educators saw their relationship with government as distant and indirect, legislation had an immediate and profound effect on their working conditions. It is government policies, regulations, standards and quality frameworks that shape working arrangements and lead to most of the working conditions highlighted as influential to educator mental health and wellbeing. A key example of this is the CCB subsidy system, which makes child care more affordable for families and influences the uptake of care. The system of reimbursement and payment can, however, have an unintended consequence of increasing educator and scheme exposure to financial insecurity and debt from unpaid fees. As described earlier, educators cannot access information about when a parents’ child care subsidy threshold has been reached, nor information about parents that have accumulated debts with other educators. Compounding dissatisfaction with financial insecurity is the low income received by many educators despite new regulations, more demanding requirements for minimum qualifications and increased quality standards and documentation.

With the new reg[ulation]s coming in and the much higher emphasis that’s being put on documentation…we’re doing the work not only of a group leader but also a director and a cleaner and a cook and we do everything that would be done in the centre but we do it all on our own and yet we’re expected to charge half of what a centre does (Educator 1).

Low wages were not, however, a uniform experience. Government deregulation of fees, an option in some schemes whereby educators can set their own fees, meant that several participants in the study ran ‘boutique’ services and earned relatively high incomes. This was possible because of the socio-economic profile of the clients and their neighbourhoods. These educators were able to offer additions such as organic food or music classes, which were valued by parents. In addition, educators that worked seven days and provided overnight care also earned higher incomes, though these gains may be offset by long working hours and lack of work flexibility. Deregulation of fees is aligned with the small business model of FDC, however, many educators noted that parents in their area could not afford to pay higher fees. Hence, while legislative changes regarding fees might be aimed at increasing the incomes of educators, in practice other constraints come in to play which prevent wage increases from being realised.

Government legislation supports the continuation of FDC as operated by individual, small businesses contracted to schemes. In 2013, around five of the approximately 450 schemes operating directly employed educators (pers comms Family Day Care Australia 2013); the remaining majority hired educators as individual contractors. Unlike other businesses, a FDC business remains one that is unable to be expanded, sold or operated independently of a scheme. The high control that schemes have over educators’ work practices sets up a quasi-employee relationship, which is a grey legislative area that is often the basis of litigation. Internationally, there is much debate over the employee/contractor ambiguity and fairness of this arrangement[64, 65]. A key informant reflected on this issue:

"It’s being promoted as a business but it’s not *truly*, it’s not respected as [as small business]. We’re being told we’re running our own small business or whatever the wording goes but it’s not really, *really* that. It’s sort of de facto employees whether we like it or not".

"As far as running our own business, it’s all a bit of a con really because there’s no possible room for expansion or great improvement in things…there’s really no great business there, there’s nothing to sell at the end of your time" (Key Informant 2).

Despite these limitations to their small business operations, many educators valued ‘being their own boss’ and the control gained from working within regulations to run the service in their own way (where possible). A drawback of the small business model was that educators have no entitlements to holiday and sick leave, or employer contributed superannuation. This lack of entitlements was a reoccurring stressor for educators. In the absence of sick leave or available relief, it was commonplace for educators to work whilst sick to avoid losing income or inconveniencing parents. While educators earning higher incomes or with employed partners could afford to take holidays, other educators spoke of the strain arising from not being able to take a break from FDC work: "I think the most stressful thing is not being able to have holidays, ‘cause I can’t afford to go on holidays. We don’t get holiday pay" (Educator 4). They also described how, as sole operators, making plans to take leave was often stressful as they dreaded inconveniencing the families using their service.

When discussing how FDC was viewed by government and society, an educator captured a common view of how educator mental health could be supported "Some respect and some credibility would be the nicest thing I think. Bit of respect is the word at the top of my list because I don’t think we get *any*" (Educator 16). A government key informant added "…whilst the industrial issues always bubble up through the research, overwhelmingly people love their jobs, they want to stay in their jobs but they feel that they’re undervalued" (Key Informant 10). Although many educators did not feel FDC was respected by or visible to government, one educator felt government esteem for child care work was demonstrated through the major reforms in early childhood education and care. Government key informants supported this view. In 2009, the Labor government began to put in place measures to standardise ECEC regulations and to increase care quality and professional standards for educators through a National Quality Agenda[66]. "The government has been really putting in a lot of effort in helping out the industry that I am in and that alone is enough to boost my confidence of how they see the importance of the job that we do, enough to make us feel good about what we do" (Educator 14). For this non-Australian born educator, the Australian government’s response to ECEC stood in stark contrast to the indifference shown by government in her country of origin. It demonstrates another way in which positive legislative and policy reform can impact the wellbeing of educators.

One educator commented that the best way government could support their mental wellbeing was in "…making sure that policies are really easy to understand and easy to follow for everyone and being consistent across the board" (Educator 7). Ambiguous quality standards and regulation documents caused considerable stress to educators and schemes alike. Educators were frustrated by the many documents and regulations that were designed and written for centre-based educators, who work in a different staffing and physical environment to FDC. They also reported that information needs to be written in accessible language for them, recognising varied educational and language backgrounds.

Why would you give us a document that is for four year qualified teachers and tell us that’s what we have to work with when yet you’re telling us that we don’t need a Certificate III as minimal qualification until 2014? … I mean we’re not silly, but when you put a document out and the wording in it, some of the wording, we’d have to look it up (Key Informant 18).

As these results suggest, there are interconnected layers of influence on educators’ mental health and wellbeing that shape FDC working conditions. Working conditions in FDC are created by educators’ interactions with social actors in their professional environment and often produced by government policy.

## Discussion

This study sought educator and key informant perspectives on how FDC working conditions influence educator mental wellbeing with a view to generating context-specific strategies for workplace mental health promotion. The research found that educators’ relationships with FDC clients, schemes and government are seen to play an integral role in their mental health and wellbeing. Relationships acted both as a psychosocial working condition and a delivery mechanism for other key working conditions that influenced mental health. As a result, relationship quality was perceived by educators to diminish, protect or promote their wellbeing. Evident throughout these relationships were repercussions and requirements related to government policies that shaped a variety of working conditions and influenced the mental health and wellbeing of educators in both positive and negative ways. The extent to which educators felt that they received fair rewards in exchange for their work was an important thread woven through their accounts of how professional relationships, working conditions, and educator mental health and wellbeing interacted. Hence, social exchange theories are revisited and explored to illustrate how the fairness of resource exchanges is fundamental to FDC educators’ mental health and wellbeing and part of collective exchanges that reinforce broader social inequities.

Consistent with the literature, positive relationships developed with children and their families were highly valued and powerful, fundamental rewards for carrying out FDC work[67, 68]. In this study, these rewards were also seen to protect and promote educator mental health and wellbeing. This is aligned with idea of ‘intrinsic’ rewards associated with child care[41] and the deeply relational nature of ECEC practice[69]. Scheme relationships were also essential to educator wellbeing as they represent a gateway to not just social support, which is a primary workplace determinant of mental health[70] but also to working conditions connected with meeting professional needs in FDC (information and services). Mental health promoting relationships with schemes were characterised by positive, respectful interactions, appropriate balance of recognition, support and autonomy for educators and the timely provision of professional information and services. These relationship characteristics align with those of trust, freedom and agency that were identified as important to educators’ experiences of resilience and ‘thriving’ in long day care settings[71]. Relationships with clients and schemes appear to constitute a crucial reward to counterbalance effort expended and buffer the effects of other job stressors. However, positive relationships alone may not be enough to sustain educators given other challenging working conditions[72], particularly those arising from government policy that has increased the demands and effort required by the educator, without appropriate increases of rewards and support. This goes some way to explaining the discordance between reports of high job satisfaction in FDC[73] alongside workforce retention problems (pers comms, FDC educators, scheme management). Although adjusting to new government regulations, frameworks, qualifications and documentation requirements was considered a job stressor, the national reform in ECEC instituted by the former Labour government promoted higher quality care and professionalism in the sector. Professionalisation of ECEC is not without criticism due to the sidelining of caring aspects in favour of education and increased monitoring and administration[74–76], nevertheless it has the potential to increase respect and recognition for ECEC work and educators’ power to improve their working conditions[74].

Significant parent-related job stressors arose primarily from insufficient support for working with parents in financially precarious positions and/or with social problems. These stressors point to the need for more equitable and increased government investment in high need families and ECEC for the benefit of families, educators and the broader community[77]. They also highlight problems with educators not getting upfront, complete payment of child care fees (which is beneficial for parents) and inadequate scheme support. Schemes occupy a powerful and potentially fraught position between government, parents and educators, which mean they have a widespread influence on educator working life and business. Poor quality relationships with schemes, as well as dissatisfaction with their provision of professional services including adequate information, were prominent job stressors. Hence, all educators in this study had moved schemes between two and four times. Relationships with schemes facilitate information exchange, which has implications not only for educator job stress but may also influence workforce capacity and practice quality[78]. Many schemes are under significant resource pressure that has increased due to ECEC reform and are likely to further strain scheme staff and lead to more stressful interactions with educators. At the macro level, many government-instituted working conditions demonstrate a lack of respect and value for FDC work, which is a common theme in FDC research[41, 79] and plays out through policies, low income and a lack of industrial protection and workplace rights.

Many working conditions perceived to be unfair and to risk educator mental wellbeing stem from educators being classed as self-employed, independent contractors and having different ‘legal and market status’ to employees[73]. Being classed as an employee grants access to a range of rights, protections and benefits that self-employed educators either miss or must self-fund, including an industrial award, superannuation, holiday and sick leave[64]. The inadequate income received by many educators in the study meant that they could not self-fund standard entitlements associated with employment. Despite the lack of entitlements, educators in this study did not express a desire to be employed by FDC schemes, should that ever be possible. In the broader educator population, however, litigation has occurred since the 1980s in Canada and in the early 1990s in Australia, to argue for employee status due to the quasi employer-employee relationships in FDC, with the intention of changing the system to ensure educators receive standard benefits of employment[64, 65]. However, governments and most FDC coordination schemes involved have been successful in circumventing or overturning decisions regarding these changes and, in turn, have avoided providing educators with the rights, protections and benefits of employees. This arrangement is unfair, if not exploitative, due to the unique business constraints on FDC (such as inability to expand), and the high control that government and schemes have over most aspects of FDC, over and above usual controls attached regulated businesses that make the relationship more in line with employment than small business structures[64, 65].

Unfair treatment of ECEC workers is also reflected in the low incomes alluded to by many of the educators in this study- this ‘pay penalty’ is consistent across all care-based profession[80]. In addition to low pay, FDC work is precarious[64] which has negative implications for workforce sustainability[81]. Precarious work, with low education and income, is associated with lower socioeconomic position, lower job control and lower job security - all detrimental to mental health and wellbeing[82]. Increases in regulatory requirements and qualifications associated with reform have not translated into higher wages for many educators, either because they are contracted to a scheme with regulated fees or because their parent clients cannot pay more for child care. These issues aside, a small subset of educators living in relatively wealthy neighbourhoods are able ‘to reap the benefits’ of small business ownership through higher incomes, making them better positioned to fund essential entitlements.

FDC educators exchange the operation of their FDC service with clients, schemes, government and society for a range of essential resources. Social exchange theories explain how the exchange of resources between educators and actors in their world influences educator mental wellbeing through the perceived fairness of exchanges and access to resources[29]. Simply put, fair exchanges support mental health and wellbeing by providing access to resources and, unfair exchanges are risky to wellbeing, depriving educators of necessary emotional, practical, symbolic and professional resources. Comparing responses with Fao and Fao’s model of key resources in social exchange, findings highlight that educators’ needs are similar to those of other working populations[30]. Specifically, results support the leading role of *equitable* exchange of five critical resources that educators and key informants believe promote mental wellbeing: high quality personal relationships; respect; services and information; and fair pay. These resources align with known determinants of population mental health that promote wellbeing and prevent mental illness that concern full social and economic participation[83]. Many of these resources can also be expected to positively influence quality of care and turnover rates. It is important to recognise that the five resource categories are interconnected. For example, low esteem has an insidious effect on relationship quality, income levels and payment, as well as the fair receipt of professional information and services.

Social exchange theories also draw attention to why certain groups are exploited while other groups have their needs not only met but also exceeded. Gender, a social determinant of health[84], has particular relevance to the imbalance of effort and reward experienced by FDC educators. FDC is a precarious, undervalued and feminised occupation, which typifies persisting gender-related power imbalances in the workplace: women tend to be segregated into lower status occupations with less job control than men[85]. Low status and respect are entwined social resources that greatly influence the power of collectives to be heard and have their needs met[86]. The FDC workforce is time-poor, isolated, largely female and attributed low status by society. This workforce also often includes working class women and those of ethnic and racial minorities, which intersects with gender[87] to further reduce the exchange power of educators. These elements present considerable challenges to gaining power to modify FDC working conditions. Despite this, strong (though ultimately unsuccessful) campaigns have been run in FDC seeking employee status[64] and employer contributed superannuation. On an individual level, educators are dependent on clients and schemes for income and operation of their FDC service, which undermines their power to advocate for change to conditions. The influence of other actors on the working conditions and mental health of FDC educators was not appreciated by many key informants who emphasised individual responsibility for educators’ mental health and wellbeing.

Overall, society benefits greatly from the social and economic participation of parents that is facilitated by poorly paid child care. It is well established and frequently recounted that ECEC is a good societal and economic investment[88, 89]. However, the ‘public goods’ provided through child care, which ensure that ‘widespread benefits accrue even to those who pay nothing’, are not reciprocated through appropriate income or good quality working conditions[90]. Despite OECD policy aims including ‘improving the working conditions and professional education of ECEC staff’, action in most countries reviewed has focused almost exclusively on attending to children’s (quality care) and families (access and affordability) needs[91]. In doing so, policy advice has emphasised greater qualifications and training, without addressing working conditions and the professional needs of educators more broadly. The potential of policymakers to increase social justice in ECEC[92] by facilitating positive changes to structural, material and symbolic working conditions that also act to protect and promote educators’ mental health is yet to be realised. To secure gains for both society and educators, it is essential to increase the value and respect for educators through fair working conditions that promote and protect their mental health and wellbeing for its own sake, in addition to creating a high quality and stable workforce. This action will involve overcoming socio-historical biases towards ‘women’s work’ and advocating for an ECEC environment that is not only good for children and families but also for educators.

### Limitations

Educators participating in the study were actively working in FDC and are not representative of those who have left the field. Most educators interviewed were recruited through schemes, which may present a limitation to the study. Those who participated independently of a scheme (snowball or educators directly recruited through email) tended to share more negative experiences, however those recruited from schemes reported a variety of perspectives of how FDC work influenced their mental health and wellbeing. Child-related job stressors, such as difficult behaviour, children with high needs and young age[40, 93] did not feature in this study, rather the role of children in supporting mental wellbeing was emphasised. As participants were questioned about the ‘hardest’ parts of FDC, it appears that other factors were more significant and, in comparison, child-related stressors were diminished or overlooked.

## Conclusions

Promoting educator mental health and wellbeing is vital not only for educator health but also for workforce stability and the provision of optimal quality care. This study presents a critical examination of how the fairness of relationships with government, FDC schemes and families using FDC services shape FDC working conditions and in turn, are perceived to influence educator mental health and wellbeing. Fair relationships were deemed to be respectful and facilitated access to social and professional support, information and services i.e. resources that reduce job stress and support wellbeing. Despite the mental health promoting role of high quality relationships, they could at best buffer the effects of unfair treatment by government concerning policies leading to low incomes, lack of entitlements and low control in how they operate their small businesses. Redressing inequities in exchange requires engagement with both FDC-specific and macro level determinants of educator mental health and wellbeing.

## Abbreviations

FDC: Family day care
ECEC: Early childhood education and care.

## References

[1] Hamre BK, Pianta RC (2004). Self-reported depression in nonfamilial caregivers: prevalence and associations with caregiver behavior in child-care settings. Early Child Res Q.

[2] Elfer P (2012). Emotion in nursery work: work discussion as a model of critical professional reflection. Early Years.

[3] Jovanovic J (2013). Retaining early childcare educators. Gend Work Organ.

[4] LaMontagne AD, Keegel T, Louie AM, Ostry A (2010). Job stress as a preventable upstream determinant of common mental disorders: a review for practitioners and policy-makers. Adv Ment Health.

[5] Butterworth P, Leach LS, Strazdins L, Olesen SC, Rodgers B, Broom DH (2011). The psychosocial quality of work determines whether employment has benefits for mental health: results from a longitudinal national household panel survey. Occup Environ Med.

[6] Mental Health Commission Canada (2010). The Shain Reports on Psychological Safety in the Workplace – A Summary.

[7] Mcdaid D, Curran C, Knapp M (2005). Promoting mental well-being in the workplace: a European policy perspective. Int Rev Psychiatry.

[8] Department of Health (2001). Making It Happen: A Guide to Delivering Mental Health Promotion.

[9] Barry MM, Jenkins R (2007). Implementing Mental Health Promotion.

[10] Davis E, Freeman R, Doherty G, Karlsson M, Everiss L, Couch J, Foote L, Murray P, Modigliani K, Owen S, Griffin S, Friendly M, McDonald G, Bohanna I, Corr L, Smyth L, Morkeseth E, Morreaunet S, Ogi M, Fukukawa S, Hinke-Ruhnau J (2012). An international perspective on family day care: comparing systems, challenges and opportunities. Austral J Early Child.

[11] **Office of Early Childhood Education: Child Care Update September 2012** In [https://education.gov.au/child-care-and-early-learning-summary]

[12] **Become an Educator** In [http://www.familydaycare.com.au]

[13] Weaver RH (2002). Predictors of quality and commitment in family child care: provider education, personal resources, and support. Early Educ Develop.

[14] Stansfeld S, Candy B (2006). Psychosocial work environment and mental health—a meta-analytic review. Scand J Work Environ Health.

[15] Nieuwenhuijsen K (2010). Psychosocial work environment and stress-related disorders, a systematic review. Occup Med.

[16] LaMontagne AD, Martin A, Page KM, Reavley NJ, Noblet AJ, Milner AJ, Keegel T, Smith PM (2014). Workplace mental health: developing an integrated intervention approach. BMC Psychiatry.

[17] Goldenhar LM, LaMontagne AD, Katz T, Heaney C, Landsbergis PA (2001). The intervention research process in occupational safety and health: an overview from the National Occupational Research Agenda Intervention Effectiveness Team. J Occup Environ Med.

[18] Hawe P, Sheil A, Riley T, Gold L (2004). Methods for exploring implementation variation and local context within a cluster randomised community intervention trial. J Epidemiol Community Health.

[19] Mazzola JJ, Schonfeld IS, Spector PE (2011). What qualitative research has taught us about occupational stress. Stress Health.

[20] Harkness AMB, Long BC, Bermbach N, Patterson K, Jordan S, Kahn H (2005). Talking about work stress: discourse analysis and implications for stress interventions. Work Stress.

[21] Kinman G, Jones F (2005). Lay representations of workplace stress: what do people really mean when they say they are stressed?. Work Stress.

[22] Kemmis S, McTaggart R, Denzin NK, Lincoln YS (2003). Participatory action research. Strategies of Qualitative Inquiry.

[23] Page KM, LaMontagne AD, Louie AM, Ostry AS, Shaw A, Shoveller JA (2013). Stakeholder perceptions of job stress in an industrialized country: implications for policy & practice. J Public Health Policy.

[24] Blaikie N (2012). Designing Social Research.

[25] Crotty M (1998). The Foundations of Social Research: Meaning and Perspective in the Research Process.

[26] Denzin NK, Lincoln YS (2003). Strategies of Qualitative Inquiry.

[27] Homans GC (1961). Social Behaviors: Its Elementary Forms.

[28] Blau PM (1964). Exchange and Power in Social Life.

[29] Walster E, Berscheid E, Walster GW (1973). New directions in equity research. J Pers Soc Psychol.

[30] Cropanzano R, Mitchell MS (2005). Social exchange theory: an interdisciplinary review. J Manag.

[31] Zafirovski M: **Some amendments to social exchange theory: a sociological perspective.** *Theor Sci* 2003.,**4**(2)**:**

[32] Siegrist J (2000). Place, social exchange and health: proposed sociological framework. Soc Sci Med.

[33] Siegrist J (1996). Adverse health effects of high-effort/low-reward conditions. J Occup Health Psychol.

[34] Moll S, Eakin JM, Franche R-L, Strike C (2013). When health care workers experience mental ill health: institutional practices of silence. Qual Health Res.

[35] Andrews Y, Newman B (2012). The value of childcare: class, gender and caring labour. Contemp Issues Early Child.

[36] Corr L, Davis E, LaMontagne AD, Waters E, Steele E (2014). Child care providers’ mental health: a systematic review of its prevalence, determinants and relationship to care quality. Int J Ment Health Promot.

[37] McInnes E, Ward C, Knight D (2010). Family day care providers’ occupational health and safety. J Health Saf Environ.

[38] Slack-Smith LM, Read AW, Darby J, Stanley FJ (2006). Health of caregivers in child care. Child Care Health Dev.

[39] Gratz RR, Claffey A (1996). Adult health in child care: health status, behaviors, and concerns of teachers, directors, and family child care providers. Early Child Res Q.

[40] Atkinson AM (1992). Stress levels of family day care providers, mothers employed outside the home, and mothers at home. J Marriage Fam.

[41] Butler JA, Modaff DP (2008). When work is home: agency, structure, and contradictions. Manag Comm Q.

[42] Baumgartner JJ, Carson RL, Apavaloaie L, Tsouloupas C (2009). Uncovering common stressful factors and coping strategies among childcare providers. Child Youth Care Forum.

[43] Groeneveld MG, Vermeer HJ, van Ijzendoorn MH, Linting M (2012). Stress, cortisol and well-being of caregivers and children in home-based child care: a case for differential susceptibility. Child Care Health Dev.

[44] Groeneveld MG, Vermeer HJ, van Ijzendoorn MH, Linting M (2010). Children’s wellbeing and cortisol levels in home-based and center-based childcare. Early Child Res Q.

[45] Curbow B, McDonnell K, Spratt K, Griffin J, Agnew J (2003). Development of the work-family interface scale. Early Child Res Q.

[46] Gerber EB, Whitebook M, Weinstein RS (2007). At the heart of child care: predictors of teacher sensitivity in center-based child care. Early Child Res Q.

[47] Kaiser J, Rogers CS, Kasper A (1993). Perceptions of well-being among child care teachers. Early Child Res Q.

[48] Boyd M (2013). "I love my work but…" the professionalization of early childhood education. Qual Rep.

[49] Goelman H, Guo H (1998). What we know and what we don’t know about burnout among early childhood care providers. Child Youth Care Forum.

[50] Kontos S, Riessen J (1993). Predictors of job satisfaction, job stress, and job commitment in family day care. J Appl Develop Psychol.

[51] Davis E, Williamson L, Mackinnon A, Cook K, Waters E, Herrman H, Sims M, Mihalopoulos C, Harrison L, Marshall B (2011). Building the capacity of family day care educators to promote children’s social and emotional wellbeing: an exploratory cluster randomised controlled trial. BMC Public Health.

[52] Guest G, Bunce A, Johnson L (2006). How many interviews are enough?: An experiment with data saturation and variability. Field Methods.

[53] Quinn-Patton M (2002). Qualitative Research and Evaluation Methods.

[54] Broffenbrenner U (1979). The Ecology of Human Development: Experiments in Nature and Design.

[55] McLaren L, Hawe P (2005). Ecological perspectives in health research. J Epidemiol Community Health.

[56] Vincent C, Braun A (2013). Being ‘fun’ at work: emotional labour, class, gender and childcare. Br Educ Res J.

[57] Fontana A, Frey JH, Denzin NK, Lincoln YS (2005). The interview: from neutral stance to political involvement. The Sage Handbook of Qualitative Research.

[58] Cook K, Gubrium J, Holstein J, McKinney K, Marvasti A (2012). Stigma and the interview encounter. Handbook of Interview Research: The Complexity of the Craft.

[59] Blaikie N (2007). Approaches to Social Enquiry.

[60] Green J, Willis K, Hughes E (2007). Generating best evidence from qualitative research: the role of data analysis. Aust N Z J Public Health.

[61] Agar M (1999). How to ask for a study in qualitatisch. Qual Health Res.

[62] Productivity Commission (2014). Child Care and Early Learning, Draft Report.

[63] **Child Care Benefit** In [http://www.humanservices.gov.au/customer/services/centrelink/child-care-benefit]

[64] Cox R (2005). Making Family Child Care Work: Strategies for Improving the Working Conditions of Family Childcare Providers.

[65] McCallum R (2008). McCallum’s Top Workplace Relations Cases: Labour Law and the Employment Relationship as Defined by Case Law.

[66] Council of Australian Governments (2009). Investing in the Early Years—A National Early Childhood Development Strategy.

[67] Curbow B, Spratt K, Ungaretti A, McDonnell K, Breckler S (2000). Development of the child care worker job stress inventory. Early Child Res Q.

[68] Skeggs B (2012). Formations of Class and Gender.

[69] Warren A (2014). Relationships for me are the key for everything’: early childhood teachers’ subjectivities as relational professionals. Contemp Issues Early Child.

[70] Michie S, Williams S (2003). Reducing work related psychological ill health and sickness absence: a systematic literature review. Occup Environ Med.

[71] Sumsion J (2004). Early childhood teachers’ constructions of their resilience and thriving: a continuing investigation. Int J Early Years Educ.

[72] Tom A (2004). Good work in Canadian childcare: complicating the love/money divide. Atlantis: Crit Stud Gend Cult Soc Justice.

[73] Anderson N, Hughes KD (2010). The business of caring: women’s self-employment and the marketization of care. Gend Work Organ.

[74] Osgood J (2006). Deconstructing professionalism in early childhood education: resisting the regulatory gaze. Contemp Issues Early Child.

[75] Cook K, Davis E, Williamson L, Harrison L, Sims M (2013). Discourses of professionalism in family day care. Contemp Issues Early Child.

[76] Woodrow C (2008). Discourses of professional identity in early childhood: movements in Australia. Eur Early Child Educ Res J.

[77] Brennan D, Adamson E (2014). Financing the future: an equitable and sustainable approach to early childhood education and care. SPRC Report 01/14.

[78] Corr L, Davis E, Cook K, Mackinnon A, Sims M, Herrman H: **Information seeking in family day care: access, quality and personal cost.** *Eur Early Child Educ Res J* 2014.,**22**(5)**:** doi:10.1080/1350293X.2014.969083

[79] Rutman D (1996). Child care as women’s work: workers’ experiences of powerfulness and powerlessness. Gend Soc.

[80] England P (2002). Wages of virtue: the relative pay of care work. Soc Probl.

[81] Clarke M, Lewchuk W, de Wolff A, King A (2007). ‘This just isn’t sustainable’: precarious employment, stress and workers’ health. Int J Law Psychiatry.

[82] Landsbergis PA, Grzywacz JG, LaMontagne AD (2012). Work organisation, job insecurity and occupational health disparities. Am J Ind Med.

[83] **The Melbourne Charter for Promoting Mental Health and Preventing Mental and Behavioural Disorders** In [http://www.vichealth.vic.gov.au/Publications/Mental-health-promotion/Melbourne-Charter.aspx]

[84] Wilkinson R, Marmot M, World Health Organization (2003). Social Determinants of Health. The Solid Facts.

[85] LaMontagne AD, Krnjacki L, Kavanagh A, Bentley R (2013). Psychosocial working conditions in a representative sample of working Australians 2001–2008: an analysis of change in inequalities over time. Occup Environ Med.

[86] Cook KS, Rice ERW, Turner JH (2001). Exchange and power: issues of structure and agency. Handbook of Sociological Theory.

[87] Duffy M (2005). Reproducing labor inequalities: challenges for feminists conceptualizing care at the intersections of gender, race and class. Gend Soc.

[88] OECD (2012). Investing in high-quality early childhood education and care (ECEC). Early Childhood and Schools.

[89] Prentice S (2009). High stakes: the "investable" child and the economic reframing of childcare. Signs.

[90] England P, Folbre N (1999). The cost of caring. Ann Am Acad Polit Soc Sci.

[91] Press F, Brennan D (2009). Trends and debates in ECEC policy. A literature review canvassing policy developments in Australia, Canada, New Zealand, Sweden and the United Kingdom. Building an International Collaboration in Early Childhood Education and Care Australian Research Alliance for Children and Youth (ARACY).

[92] Press F, Skattebol J (2007). Early childhood activism, minor politics and rescuscitating vision: a tentative foray into the use of ‘intersections’ to influence early childhood policy. Contemp Issues Early Child.

[93] de Schipper EJ, Riksen-Walraven JM, Geurts SAE (2007). Multiple determinants of caregiver behavior in child care centers. Early Child Res Q.

[CR94] The pre-publication history for this paper can be accessed here:http://www.biomedcentral.com/1471-2458/14/1214/prepub

